# Clinicopathologic implication of microRNA-197 in diffuse large B cell lymphoma

**DOI:** 10.1186/s12967-018-1537-0

**Published:** 2018-06-11

**Authors:** Jeong Mi Yang, Ji-Young Jang, Yoon Kyung Jeon, Jin Ho Paik

**Affiliations:** 1 Department of Pathology, Seoul National University Bundang Hospital, 300 Gumi-dong, Bundang-gu, Seongnam, Gyeonggi South Korea; 2 Department of Pathology, Seoul National University Hospital, Seoul, South Korea; 3 Department of Pathology, Seoul National University College of Medicine, Seoul, South Korea; 4 BIOINFRA Life Science Inc., Seoul, South Korea

**Keywords:** microRNA, miR-197, Diffuse large B cell lymphoma, Chemosensitivity, Apoptosis

## Abstract

**Background:**

Diffuse large B cell lymphoma (DLBCL) contains heterogeneous subtypes with various molecular dysregulation at the gene, protein and microRNA levels. Compared with the GCB subtype, the non-germinal center B-like (non-GCB)/activated B cell-like (ABC) subtype exhibits frequent progression despite standard immunochemotherapy. We aimed to investigate the effects of miR-197 on the progression and chemosensitivity of DLBCL with respect to the GCB and non-GCB/ABC subtypes.

**Methods:**

To screen distinctively expressed microRNAs, microRNA expression patterns were analyzed in 10 DLBCL cases by microarray chip assays. Using quantitative real-time polymerase chain reaction (qRT-PCR), associations between miR-197 expression levels and clinicopathologic variables were investigated in 51 DLBCL tissue samples. The effects of miR-197 on doxorubicin chemosensitivity were investigated using the OCI-Ly1 and SUDHL9 cell lines.

**Results:**

MicroRNA expression profiling by hierarchical clustering revealed that miR-197 was one of the distinctively expressed microRNAs in DLBCL. Quantitative analysis using qRT-PCR revealed that miR-197 levels were not correlated with clinicopathologic variables, including the international prognostic index, but low miR-197 levels were significantly associated with lymphoma progression defined by refractoriness, relapse or death in the rituximab plus cyclophosphamide, doxorubicin, vincristine, and prednisone (R-CHOP)-treated subgroup (n = 43; p = 0.004). Among the three molecular groups, i.e., the GCB, non-GCB/miR-197^low^ and non-GCB/miR-197^high^ groups, progression was most frequently observed in the non-GCB/miR-197^low^ group in the full cohort (p = 0.013) and the R-CHOP cohort (p = 0.008). In survival analysis, low miR-197 levels were independently predictive of shorter progression-free survival in the R-CHOP cohort (p = 0.031; HR = 27.9) and the non-GCB subgroup (p = 0.037; HR = 21.5) but not in the GCB subgroup. Using SUDHL9 (ABC type) and OCI-Ly1 (GCB type) cells, the effects of doxorubicin on reducing cell viability were enhanced by miR-197 transfection. In apoptosis assays, miR-197 transfection enhanced doxorubicin-induced apoptosis in SUDHL9 cells but not in OCI-Ly1 cells, suggesting a chemosensitizing effect of miR-197 in ABC DLBCL.

**Conclusions:**

These results suggest the role of miR-197 as a biomarker with potential therapeutic implications.

**Electronic supplementary material:**

The online version of this article (10.1186/s12967-018-1537-0) contains supplementary material, which is available to authorized users.

## Background

Diffuse large B-cell lymphoma (DLBCL) is the most common type of malignant lymphoma, accounting for 30–40% of non-Hodgkin lymphoma cases [1]. DLBCL can be classified into two cell-of-origin (COO) subtypes, i.e., germinal center B cell (GCB) and activated B cell (ABC) types, which exhibit different gene expression profiles and clinical outcomes [2–6]. ABC DLBCL is recognized as a more aggressive subtype despite treatment with standard chemotherapy with rituximab plus cyclophosphamide, doxorubicin, vincristine, and prednisone (R-CHOP) [5]. Non-GCB DLBCL based on Hans and related algorithms using immunohistochemistry was inferior to GCB DLBCL regarding survival. In addition, non-GCB DLBCL is thought to represent ABC DLBCL with a defined gene expression signature [2, 3, 6] despite several inconsistent studies [7–9]. Although the clinical outcome of DLBCL can been distinctively improved with R-CHOP therapy, approximately 1/3 of DLBCL patients still experience relapse or refractoriness [10]. In this context, various studies have been performed to uncover and validate useful biomarkers to predict chemotherapeutic responses and clinical outcomes of DLBCL patients at the gene and protein levels as well as the post-transcriptional level of gene expression as modulated by microRNAs [11–13].

Doxorubicin is an anticancer drug in the anthracycline family and is used to treat various types of malignancies, including breast carcinoma, soft tissue sarcomas, acute leukemia and non-Hodgkin lymphomas [14–16]. Several complex mechanisms, including topoisomerase II inhibition, DNA intercalation, free radical generation, and the activation of several transcription factors, including cAMP responsive element binding protein 3-like 1 (CREB3L1), have been proposed to explain the antitumor activity of doxorubicin [17–19]. These biologic processes are regulated by many genes, proteins and microRNAs, and the response to this drug depends on the cellular context as it contains various regulatory molecules at the protein, gene and microRNA levels.

MicroRNAs are small non-coding RNAs composed of 20–24 nucleotides [20]. MicroRNAs are involved in many important biological processes, including cell proliferation, differentiation and apoptosis [21, 22], which might also be associated with critical oncogenic or tumor-suppressive pathways in neoplastic disease [23–25]. Therefore, the roles of microRNAs have been investigated in various types of solid and hematologic tumors, including DLBCL [26, 27], where distinct expression profiles were shown for GCB and ABC subtypes [28–30]. Several microRNAs are known to influence the sensitivity of tumor cells to anticancer drugs, consequently affecting the clinical outcomes of patients with hepatocellular carcinoma and chronic lymphocytic leukemia [31, 32]. MiR-451 is associated with doxorubicin resistance in breast cancer cells [33]. MiR-21 and miR-34a are regulators of chemosensitivity in DLBCL [34, 35]. Furthermore, miR-18a, miR-181a, miR-222, miR-199a and miR-497 predict overall and/or progression-free survival of DLBCL patients [36, 37].

Another important regulator of cancer-associated signaling in various solid and hematologic neoplasms is miR-197, which is involved in various pathways of cancer progression and chemoresistance depending on cell type and cellular context. MiR-197 regulates the response to 5-fluorouracil treatment in gastric and colorectal cancers via targeting mitogen-activated protein kinase 1 and thymidylate synthase, respectively [38, 39]. MiR-197 suppresses the p53-dependent apoptosis pathway in lung cancers [40] but induces apoptosis in myeloma cells via targeting Mcl-1 [41]. However, the role of miR-197 in DLBCL has not clearly been elucidated to date with respect to prognosis and chemoresistance.

In the present study, we aimed to investigate the effects of miR-197 in response to chemotherapy in DLBCL patients and to determine the possibility of miR-197 as an adjuvant therapeutic agent to enhance the effect of doxorubicin in DLBCL patients, especially those with the ABC subtype.

## Methods

### Patients and human tissue specimens

A total of 51 patients diagnosed with DLBCL at Seoul National University Hospital from 2006 to 2012 and Seoul National University Hospital from 2003 to 2012 were enrolled. The patients’ histologic slides and clinical medical records were retrospectively reviewed. According to the 2008 WHO classification of lymphoma, primary CNS lymphoma and mediastinal large B-cell lymphoma were excluded [42]. All DLBCL cases were divided into GCB and non-GCB subtypes using the Hans algorithm based on immunohistochemical findings [6]. The follow-up duration ranged from 0.5 to 64 months (median, 20.7 months). Twelve patients (24%) had progression and died at the time of analysis. Progression was defined as refractoriness, relapse or death. Progression-free survival (PFS) was defined as the time from first treatment to lymphoma progression, including death of any cause [43]. The institutional review board approved this study.

### Cell lines and reagents

SUDHL9 and OCI-LY1 are human DLBCL cell lines. SUDHL9 cells were cultured in Roswell Park Memorial Institute 1640 (RPMI 1640) supplemented with 10% inactivated fetal bovine serum (WELGENE, Seoul, South Korea) and 1% antibiotic–antimycotic solution (WELGENE) in a humidified atmosphere with 5% CO_2_ in air at 37 °C. OCI-LY1 cells were cultured in Iscove’s Modified Dulbecco’s Medium (WELGENE) supplemented with 20% inactivated fetal bovine serum and 1% antibiotic–antimycotic solution. Doxorubicin (Cat no. D1515, Sigma-Aldrich, St. Louis, MO, USA) was purchased from Sigma-Aldrich.

### RNA extraction from formalin-fixed tissues and cell lines

Total RNA was extracted from FFPE tissue samples using a RecoverAll™ Total Nucleic Acid Isolation for FFPE kit (Cat no. AM197, Applied Biosystems, Foster City, CA, USA) according to the manufacturer’s protocol and was stored at − 80 °C until the time of use after measuring the concentration with a NanoDrop 2000 spectrophotometer (Thermo Fisher Scientific, Waltham, MA, USA). Total RNA was extracted from DLBCL cell lines using TRI REAGENT (Cat no. TR118, Molecular Research Center, Cincinnati, OH, USA). MicroRNA was evaluated by qRT-PCR using Mir-X microRNA first strand synthesis and the SYBR qRT-PCR kit (Cat no. 638313, Clontech Laboratories, Mountain View, CA, USA). U6 served as an internal control.

### Screening of microRNAs using a microarray chip test in DLBCL tissues

To screen distinctly expressed microRNAs in DLBCL, microRNA expression profiles in 10 formalin-fixed paraffin-embedded (FFPE) DLBCL tissue samples were investigated using a peptide nucleic acid (PNA)-based microRNA expression profiling kit (PANArray™, #PM-1001, version 1, Panagene, Daejeon, South Korea) containing 135 probes for cancer-related microRNAs according to the manufacturer’s instruction. U6 was included in the microarray slide as an internal control for normalization. The average value of the duplicate tests was used for expression profiling analysis. In this chip-based screening study, 73 microRNAs significantly expressed in DLBCL tissues were included in hierarchical clustering analysis of the agglomerative type (average linkage clustering). The DLBCL cases and microRNAs were reordered and clustered using the ‘heatmap.2’ function in the ‘gplots’ package in R using Euclidean measurements to obtain a distance matrix and the complete average agglomeration method [44].

### Quantitative real-time polymerase chain reaction

Levels of hsa-miR-197 (cat no. 4427975, Applied Biosystems) were determined by qRT-PCR using TaqMan Universal PCR Master Mix and the TaqMan microRNA Reverse Transcription kit (Cat no. 4366596, Applied Biosystems), and U6 snRNA served as an internal control. The expression levels of the microRNAs were compared using 11 normal tonsil FFPE tissues as normal controls. The relative level of miR-197 in OSCC was calculated as 2^−ΔΔCt^, where ΔCt = Ct (miR-197) − Ct (U6) and ΔΔCt = ΔCt (tumor) − ΔCt (normal).

### Cell culture and transfection of microRNA mimics

SUDHL9 and OCI-LY1 cells were seeded at a density of 3 × 10^5^ cells/well in 12-well plates and were cultured in Opti-MEM (Gibco, Grand Island, NY, USA). For transfection, the transfection agent Lipofectamine 2000 (Invitrogen, Waltham, MA, USA) and 50 and 100 nM microRNAs synthesized at GenePharma (Shanghai, China) were added to the cultured cells, and complementary media was added after 6 h.

### Cell viability assay

SUDHL9 and OCI-LY1 cells were seeded at a density of 3 × 10^5^ cells/well in 12-well plates. At 24 h post-transfection, various concentrations (0–100 nM for SUDHL9; 0–200 nM for OCI-LY1) of doxorubicin were added to the cells, resulting in a total volume of 1000 µl/well. At 48 h after doxorubicin treatment, 200 µl of treated cells were transfer to 96-well plates. Twenty microliters of EX-CYTOX (EX-3000, DOGEN Bio., Seoul, South Korea) agent was added to each well. Then, the cells were maintained in an incubator with 5% CO_2_ at 37 °C for 2–3 h. The absorbance at 450 nm, which is indicative of cell viability, was detected. All reactions were analyzed in triplicate.

### Apoptosis assay

SUDHL9 and OCI-LY1 cells were treated with the same transfection conditions. Twenty-four hours post-transfection, SUDHL9 cells were treated with 25 and 50 nM doxorubicin for 48 h, and OCI-LY1 cells were treated with 50, 100 and 200 nM doxorubicin for 72 h. For analysis of apoptosis, cells were stained with 3 µl of Annexin V and 1.5 µl of PI for 10 min at RT in the dark using the FITC Annexin V Apoptosis Detection Kit I (556547, BD Biosciences, San Jose, CA, USA). All reactions were analyzed in triplicate using BD flow cytometry (BD FACScalibur, BD Biosciences).

### Statistical analysis

The microRNA expression data obtained from microarray chip assays for ten cases of DLBCL were analyzed by hierarchical clustering with dendrograms using R package 3.1.2 (gplots). Dot plots for comparison of microRNA expression levels between subgroups were also generated by R package 3.1.2 (ggplot2 and beeswarm). Other statistical analyses were performed using SPSS 21.0 (IBM, Armonk, NY, USA). All experiments were independently performed in triplicate. Independent sample t-tests were used for comparison of microRNA expression levels. Survival data were analyzed using Kaplan–Meier analysis with log-rank tests and Cox proportional hazard models. p-values less than 0.05 were considered statistically significant. MiR-197 expression was categorized as high or low expression based on the cutoff point exhibiting maximum χ^2^ (minimum p-value) as a prognostic factor [45]. This method employs a systematic search of almost all observed values as the candidate cutoff point and selects the value associated with a maximum χ^2^ (or minimum p value) as the final cutoff point [45].

## Results

### Screening of distinctively expressed microRNAs and identification of miR-197 in DLBCL

**Fig. 1:**
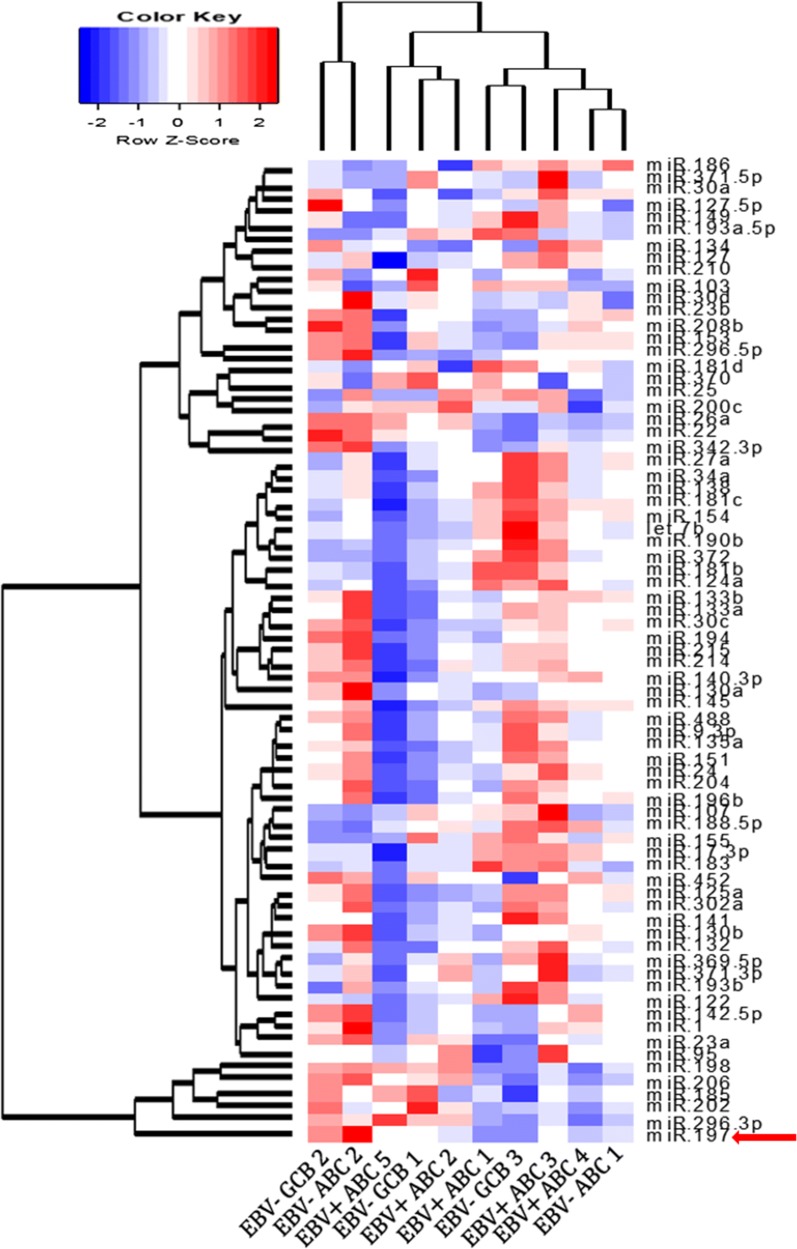
Heatmap with dendrograms generated by hierarchical clustering in DLBCLs. Based on a hierarchical clustering algorithm, six microRNAs (miR-197, miR-296-3p, miR-202, miR-185, miR-206 and miR-198) were distinctively clustered irrespective of DLBCL subtypes. MiR-197 is marked with a red arrow

By analyzing expression profiling using the cancer-related microRNA microarray platform, six microRNAs (miR-197, miR-296-3p, miR-202, miR-185, miR-206 and miR-198) were recognized to form a distinctive cluster in DLBCLs, as shown in the heatmap with dendrograms generated using a hierarchical clustering algorithm (Fig. 1 and Additional file 1: Figure S1). Among the six microRNAs, miR-197 exhibited the most distinctive pattern in the cluster, and miR-197 expression levels exhibited wide ranges among the various subtypes of DLBCLs. Therefore, we focused on the quantitative analysis of miR-197 to investigate its clinicopathologic association in DLBCLs.

### Clinicopathologic features of diffuse large B cell lymphoma patients

The clinicopathologic features of 51 cases of DLBCL are presented in Table 1. Among the 51 cases of DLBCL, 22% (11/51) were EBV positive. Old age (≥ 60 years), male patients, extranodal cases and low international prognostic index (IPI) were common features, accounting for approximately 60% of cases. Based on the Hans algorithm, 73% (37/51) of cases were classified as the non-GCB type, which included all 11 cases of EBV-positive DLBCL. R-CHOP therapy was administered to 84% (43/51) of patients.

**Table 1:** Clinicopathologic characteristics of diffuse large B cell lymphoma patients

Variables	Full cohort (n = 51)	R-CHOP cohort (n = 43)
	Number of cases (%)	Number of cases (%)
Age, years		
Mean (range)	59.6 (21–85)	
< 60	21 (41%)	17 (40%)
≥ 60	30 (59%)	26 (60%)
Sex		
Male	30 (59%)	24 (56%)
Female	21 (41%)	19 (44%)
Primary site		
Nodal	18 (35%)	14 (33%)
Extranodal	33 (65%)	29 (67%)
Ann Arbor stage		
I, II	27 (53%)	23 (54%)
III, IV	24 (37%)	20 (46%)
IPI group^a^		
Low (0–2)	30 (60%)	27 (63%)
High (3–5)	20 (40%)	16 (37%)
B symptoms^a^		
Absent	31 (62%)	29 (67%)
Present	19 (38%)	14 (33%)
ECOG PS^a^		
0, 1	40 (82%)	37 (86%)
≥ 2	9 (18%)	6 (14%)
LDH^a^		
Normal	21 (42%)	19 (44%)
Elevated	29 (58%)	24 (56%)
BM involvement^a^		
Absent	42 (91%)	38 (93%)
Present	4 (9%)	3 (7%)
Number of extranodal sites^a^		
0, 1	40 (80%)	33 (77%)
≥ 2	10 (20%)	10 (23%)
EBER		
Negative	40 (78%)	36 (84%)
Positive	11 (22%)	7 (16%)
Hans classification		
GCB	14 (27%)	12 (28%)
Non-GCB	37 (73%)	31 (72%)
Treatment		
Rituximab + CHOP	43 (84%)	43 (100%)
Rituximab + others	1 (2%)	0 (0%)
Surgery only	2 (4%)	0 (0%)
No Tx	5 (10%)	0 (0%)

*IPI* international prognostic index, *ECOG PS* Eastern Cooperative Oncology Group Performance Status, *LDH* lactate dehydrogenase, *BM* bone marrow, *EBER* EBV-encoded RNA, *GCB* germinal center B-like, *CHOP* cyclophosphamide, doxorubicin, vincristine, and prednisone

^a^These variables exclude missing values

### MiR-197 expression levels according to different clinicopathologic groups

**Fig. 2:**
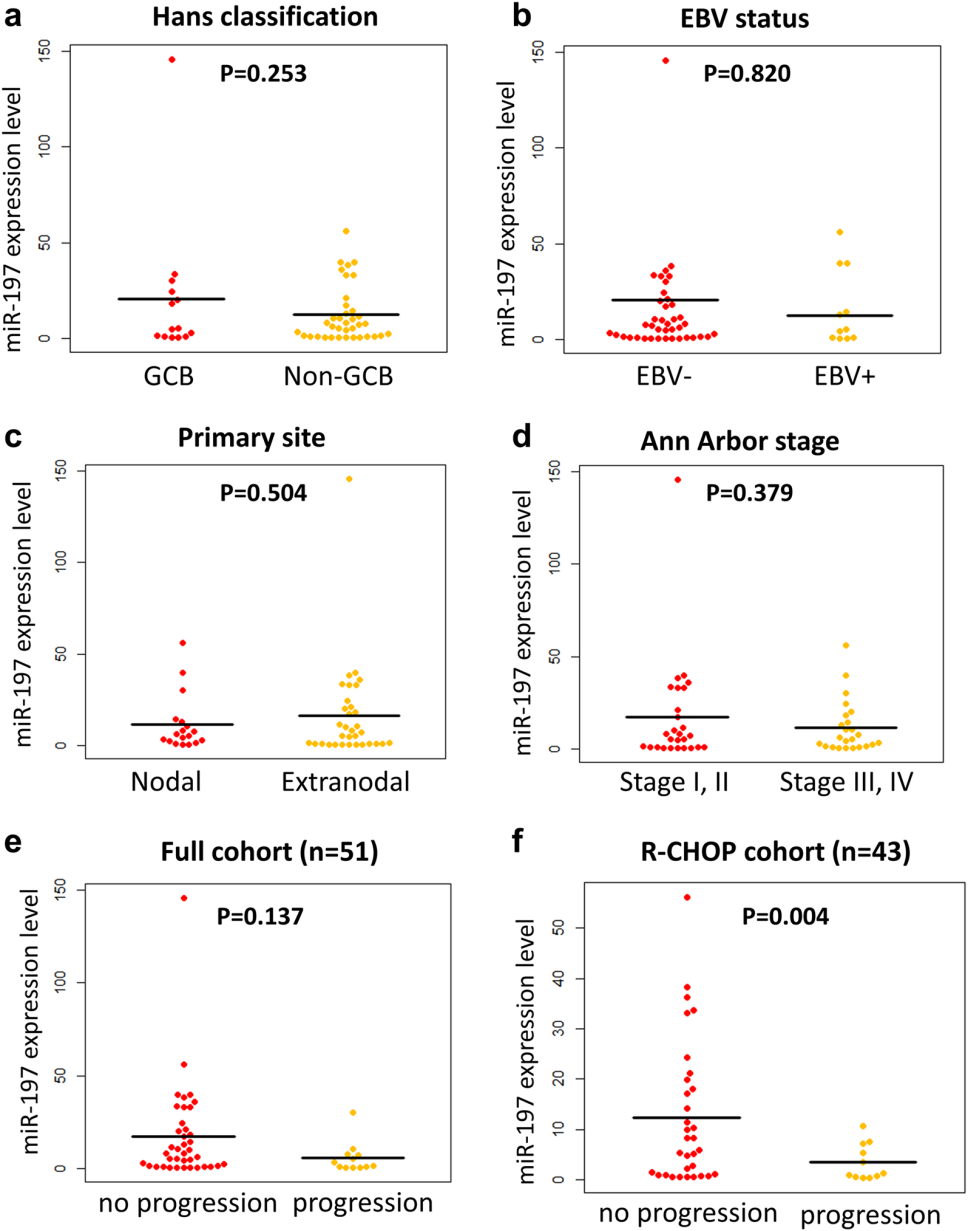
Relative miR-197 expression level based on clinicopathologic variables and progression status. Relative miR-197 expression levels did not differ based on Hans classification (**a**), EBV infection (**b**), primary site (**c**) or Ann Arbor stage (**d**) in the full cohort of diffuse large B-cell lymphoma (n = 51). MiR-197 levels tended to be reduced in the progression group compared with the no progression group in the full cohort (n = 51) (**e**). In the R-CHOP cohort (n = 43), miR-197 was significantly reduced in the progression group (**f**)

As shown in Fig. 2a–d and Table 2, miR-197 expression levels were compared between various subgroups and clinicopathologic factors of DLBCL, i.e., EBV-positive vs. EBV-negative cases; GCB vs. non-GCB/ABC type; and nodal vs. extranodal cases. Based on comparisons of mean values using independent t-tests, miR-197 expression levels were not significantly associated with EBV status, nodal/extranodal, stages (I, II vs. III, IV) and IPI (0–2 vs. 3–5). The non-GCB subtype tended to have low miR-197 expression levels, but the result did not achieve statistical significance (p = 0.253).

**Table 2:** Comparison of miR-197 expression levels according to clinicopathologic characteristics in diffuse large B cell lymphoma patients

Variables	Full cohort (n = 51)		R-CHOP cohort (n = 43)	
	Mean ± SD	p value	Mean ± SD	p value
Age, years		0.415		0.357
< 60	18.2 ± 32.6		7.8 ± 12.0	
≥ 60	12.0 ± 13.7		11.6 ± 13.4	
Sex		0.603		0.353
Male	16.0 ± 27.7		8.5 ± 10.4	
Female	12.5 ± 15.4		12.2 ± 15.4	
Primary site		0.504		0.595
Nodal	11.5 ± 15.3		8.6 ± 14.3	
Extranodal	16.2 ± 26.8		10.8 ± 12.3	
Ann Arbor stage		0.379		0.731
I, II	17.3 ± 29.2		10.7 ± 12.8	
III, IV	11.5 ± 14.1		9.4 ± 13.2	
IPI group^a^		0.664		0.985
Low (0–2)	11.2 ± 13.0		10.1 ± 12.3	
High (3–5)	13.0 ± 15.6		10.1 ± 14.1	
B symptoms^a^		0.737		
Absent	15.7 ± 27.4		10.4 ± 12.2	
Present	13.4 ± 15.6		9.5 ± 14.4	
ECOG PS^a^		0.886		0.310
0, 1	12.0 ± 13.8		10.9 ± 13.5	
≥ 2	12.8 ± 16.0		5.1 ± 5.9	
LDH^a^		0.466		0.785
Normal	10.2 ± 13.2		9.5 ± 12.6	
Elevated	13.2 ± 14.6		10.6 ± 13.3	
BM involvement^a^		0.561		0.452
Absent	11.0 ± 12.5		9.1 ± 11.2	
Present	19.2 ± 24.8		24.1 ± 27.9	
Number of extranodal sites^a^		0.182		0.582
0, 1	12.9 ± 15.0		10.7 ± 14.0	
≥ 2	8.1 ± 8.0		8.1 ± 8.0	
EBER		0.820		0.804
Negative	14.1 ± 24.4		9.9 ± 11.2	
Positive	16.0 ± 19.8		11.2 ± 20.4	
Hans classification		0.253		0.833
GCB	20.7 ± 38.0		9.4 ± 11.5	
Non-GCB	12.2 ± 14.6		10.4 ± 13.5	

*IPI* international prognostic index, *ECOG PS* Eastern Cooperative Oncology Group Performance Status, *LDH* lactate dehydrogenase, *BM* bone marrow, *EBER* EBV-encoded RNA, *GCB* germinal center B-like

^a^These variables exclude missing values

### Differential expression levels of miR-197 based on progression status

MiR-197 expression levels were compared between the patients who had progression, i.e., refractoriness, relapse, or death, and those who did not exhibit progression during the follow-up period (Fig. 2e, f). Patients who progressed exhibited significantly low miR-197 levels in the R-CHOP-treated cohort (p = 0.004; n = 43), whereas the difference was not significant in the full cohort (p = 0.137; n = 51).

### Associations among progression status, GCB/non-GCB subtype and miR-197 expression

**Fig. 3:**
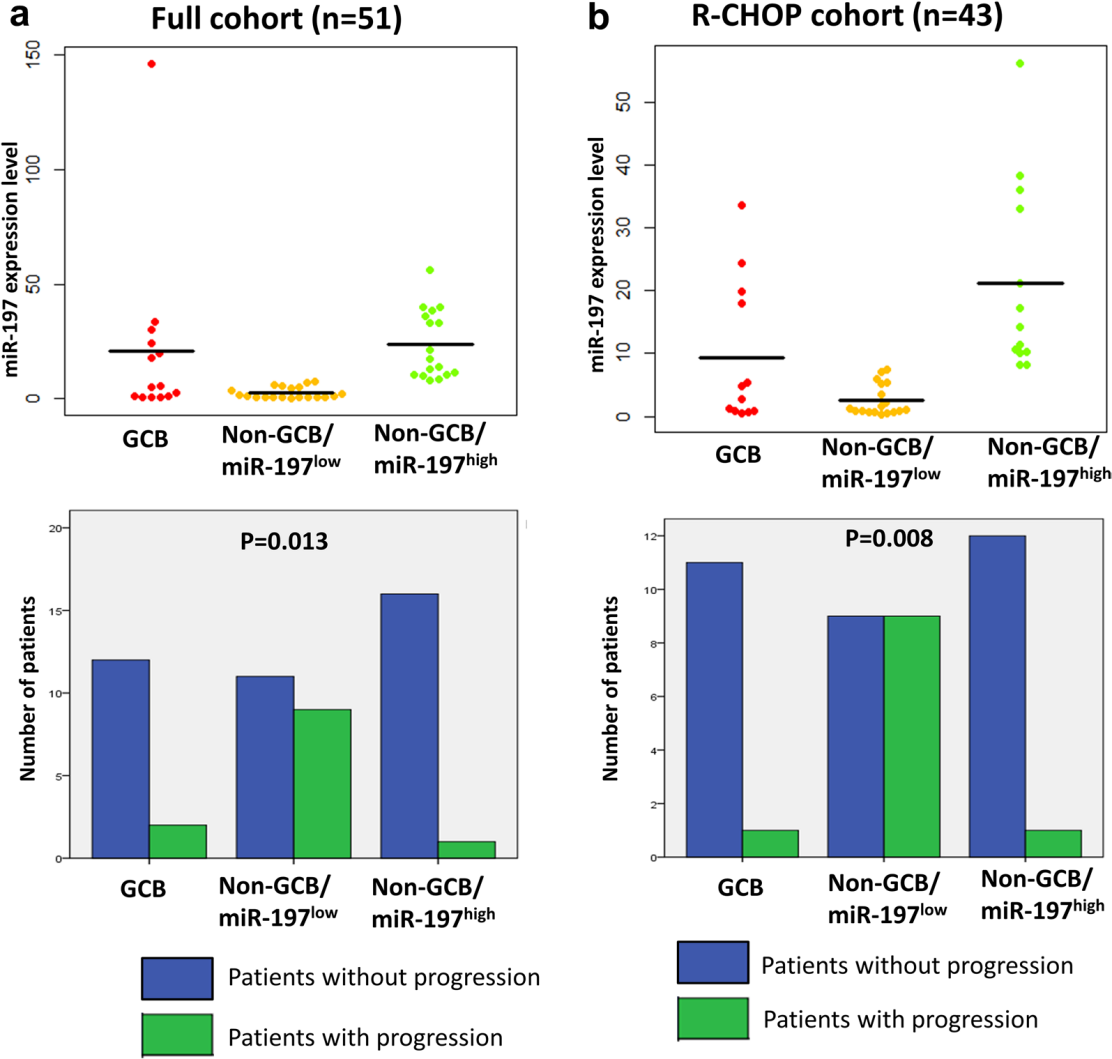
Comparison of relative miR-197 expression levels and frequency of patients with progression in the GCB, non-GCB/miR-197^low^ and non-GCB/miR-197^high^ subgroups of diffuse large B-cell lymphoma. Compared with the GCB and non-GCB/miR-197^high^ subgroups exhibiting similar levels of miR-197 expression, the non-GCB/miR-197^low^ subgroup exhibited low miR-197 levels and high progression rates in full cohort (**a**) and R-CHOP cohort (**b**)

Considering the significant effects of miR-197 on progression within the non-GCB subgroup, we analyzed the frequencies of progression among the three molecular groups, i.e., GCB, non-GCB/miR-197^low^ and non-GCB/miR-197^high^ groups. As shown in Fig. 3, progression was most frequently noted in the non-GCB/miR-197^low^ group in the full cohort (p = 0.013) and the R-CHOP cohort (p = 0.008).

### Survival analysis of progression-free survival based on miR-197 expression levels

**Fig. 4:**
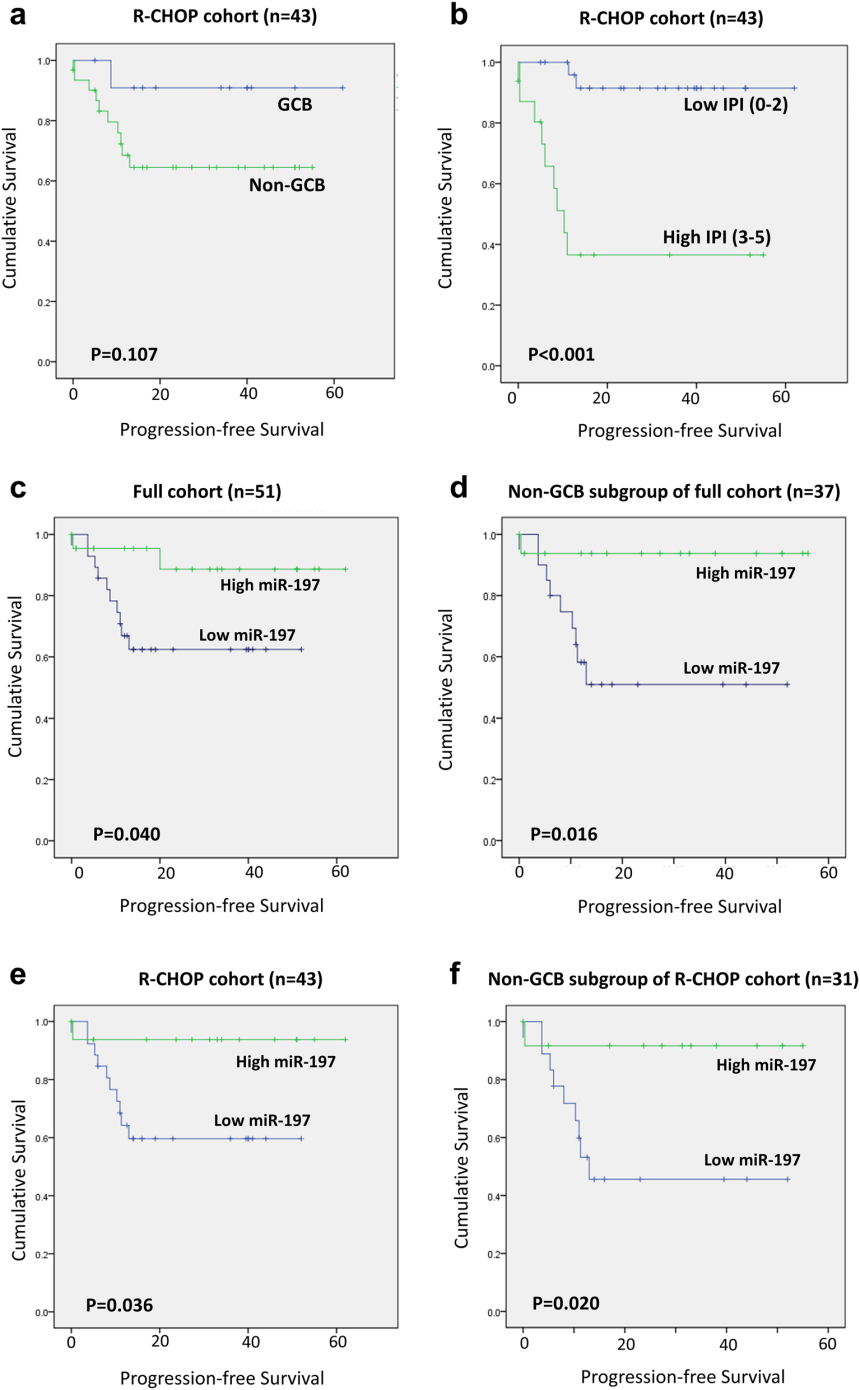
Survival curves for progression-free survival in diffuse large B-cell lymphoma. The non-germinal center B-like (non-GCB) subtype tended to exhibit shorter progression-free survival (PFS) compared with the GCB subtype (**a**), whereas a high international prognostic index (IPI) was a significant predictor of shorter PFS (**b**) in the R-CHOP cohort (n = 43). Low miR-197 levels were also associated with shorter PFS in the full cohort (n = 51) (**c**) and its non-GCB subgroup (n = 37) (**d**) as well as the R-CHOP cohort (n = 43) (**e**) and its non-GCB subgroup (n = 31) (**f**)

Given the close relationship between progression status and miR-197 expression level, the effects of miR-197 and various clinicopathologic variables on PFS were analyzed using the Kaplan–Meier method with log-rank tests. As shown in Fig. 4, the GCB type tended to have a good prognosis without statistical significance (p = 0.107; Fig. 4a), whereas low IPI was significantly associated with longer PFS (p < 0.001; Fig. 4b). The miR-197^low^ group exhibited reduced PFS compared with the miR-197^high^ group in the full cohort (n = 51; n = 0.040; Fig. 4c) and its non-GCB subgroup (n = 37; p = 0.016; Fig. 4d) as well as the R-CHOP cohort (n = 43; p = 0.036; Fig. 4e) and its non-GCB subgroup (n = 31; p = 0.020; Fig. 4f). In contrast, the prognostic significance of miR-197 was not evident in the GCB subgroup of the full cohort or R-CHOP cohort (p > 0.05).

### Progression-free survival analysis based on miR-197 expression levels and clinicopathologic prognostic factors in the R-CHOP cohort

Table 3 summarizes the prognostic factors predicting PFS using univariate and multivariate analysis in the R-CHOP cohort (n = 43) and ABC subgroup (n = 31). In the R-CHOP cohort, miR-197 was associated with PFS in univariate analysis. Other prognostic factors included IPI, B symptoms, bone marrow involvement, stage, performance status, lactate dehydrogenase (LDH), and extranodal site number. Multivariate analysis revealed that IPI (p = 0.009; HR = 10.0), miR-197 (p = 0.031; HR = 27.9), and bone marrow involvement (p = 0.038; HR = 15.4) predicted PFS. In the non-GCB subgroup, univariate analysis revealed similar prognostic factors, including miR-197 and other clinicopathologic factors. Multivariate analysis incorporating miR-197, IPI and bone marrow involvement revealed that miR-197 (p = 0.037; HR = 21.5) and bone marrow involvement (p = 0.046; HR = 13.5) were significant prognostic factors.

**Table 3:** Univariate and multivariate survival analysis of clinicopathologic variables and miR-197 expression in diffuse large B cell lymphoma patients

Clinicopathologic variables	R-CHOP cohort, n = 43			ABC R-CHOP cohort, n = 31		
	UA	MA		UA	MA	
	p	p	HR [95% CI]	p	p	HR [95% CI]
miR-197						
Low vs. high	0.036	0.031	27.9 [1.4–569.0]	0.025	0.037	21.5 [1.2–382.6]
IPI						
High (3–5) vs. low (0–2)	< 0.001	0.009	10.0 [1.8–56.1]	0.001	0.055	5.1 [1.0–26.6]
B symptoms						
Presence vs. absence	0.024	0.844	1.2 [0.3–4.8]	0.059		
Bone marrow involvement						
Presence vs. absence	0.006	0.038	15.4 [1.2–203.3]	0.029	0.046	13.5 [1.1–173.1]
Stage^a^						
I–II vs. III–IV	0.004			0.012		
Performance status^a^						
≥ 2 vs. 0–1	< 0.001			< 0.001		
LDH^a^						
Elevated vs. normal	0.005			0.005		
Number of extranodal sites^a^						
≥ 2 vs. 0–1	< 0.001			0.002		

*UA* univariate analysis, *MA* multivariate analysis, *95% CI* 95% confidence interval, *IPI* international prognostic index, *LDH* lactate dehydrogenase

^a^These variables were not included in multivariate analysis due to their close relationship with IPI

### Cell viability assay after doxorubicin and miR-197 transfection treatment in DLBCL cell lines

**Fig. 5:**
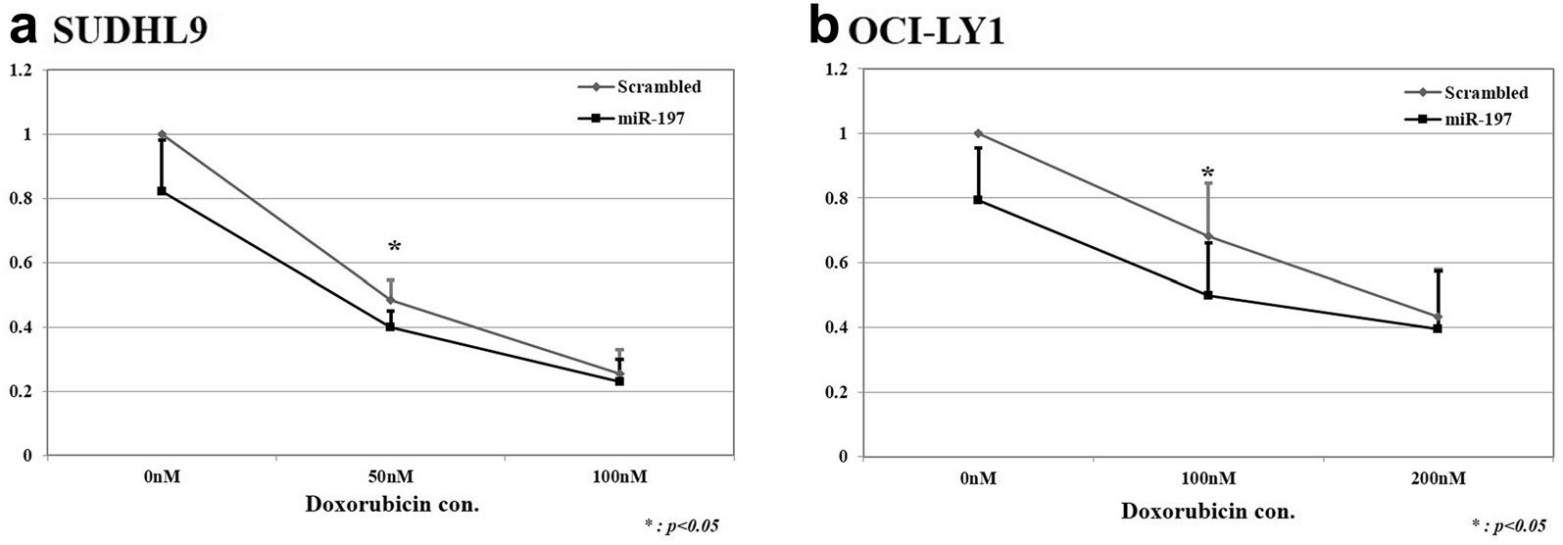
Cell viability assay results for doxorubicin treatment and miR-197 transfection in diffuse large B-cell lymphoma cell lines. Upon treatment with a series of doxorubicin concentrations in diffuse large B-cell lymphoma cells of non-germinal center/activated B cell-like (non-GCB/ABC; SUDHL9) phenotype and GCB origin (OCI-LY1), cell viability was significantly reduced in the miR-197 transfection group compared with that in the scrambled control group. SUDHL9, an ABC DLBCL cell line, exhibited significant miR-197-transfection-induced reduction in cell viability at lower concentrations of doxorubicin (**a**) compared with OCI-Ly1, a GCB DLBCL cell line (**b**)

Given the results from clinical samples demonstrating that miR-197 influenced the progression status and PFS, we hypothesized that miR-197 may be associated with chemoresistance in DLBCL tumor cells. Next, we selected two cell lines with low miR-197 expression to investigate the role of miR-197 in DLBCL cell lines treated with doxorubicin, a key tumoricidal component of the R-CHOP chemotherapeutic regimen. SUDHL9, an ABC type DLBCL cell line, and OCI-Ly1, a GCB type DLBCL cell line, cells were treated with doxorubicin with or without miR-197 mimic transfection. Upon transfection with the miR-197 mimic, cell viability significantly decreased after doxorubicin treatment in SUDHL9 and OCI-Ly1 cells compared with that in the scrambled control group (Fig. 5).

### Apoptosis assay

**Fig. 6:**
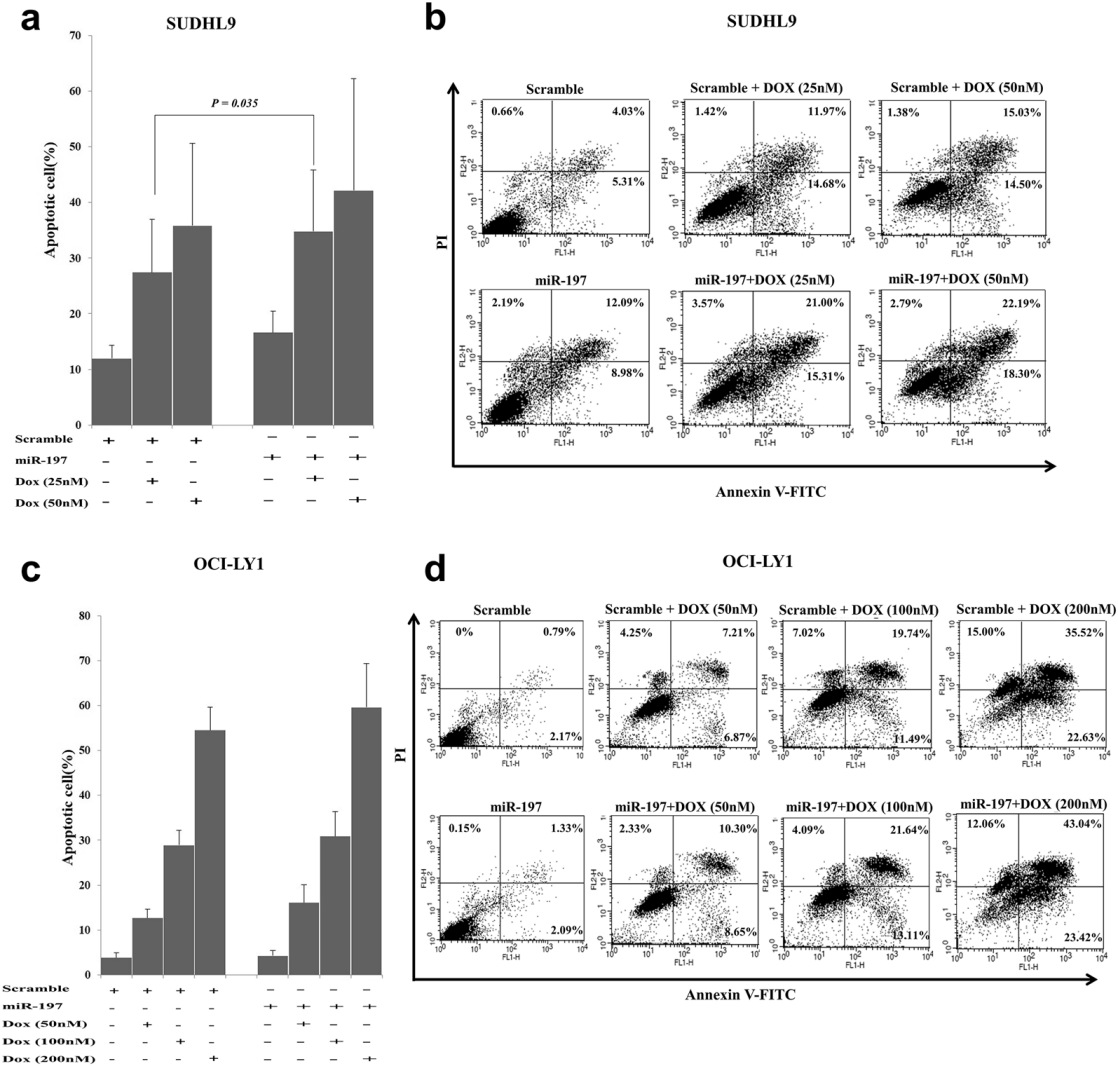
Apoptosis assay results for doxorubicin treatment and miR-197 transfection in diffuse large B-cell lymphoma cell lines. Measurement of apoptotic cell proportions upon transfection of miR-197 in SUDHL9, an activated B cell-like diffuse large B-cell lymphoma (ABC DLBCL) cell line, revealed the induction of a significant chemosensitizing effect to 25 nM doxorubicin treatment as shown in the bar graph (**a**) and representative flow cytometry plot (**b**), whereas no significant difference was observed in OCI-LY1, a germinal center B-like DLBCL cell line, as shown in the bar graph (**c**) and representative flow cytometry plot (**d**)

In the apoptosis assay with Annexin V and propidium iodide (PI) staining using flow cytometry, the effects of miR-197 differed between SUDHL9 and OCI-Ly1 cells (Fig. 6). In SUDHL9 cells, miR-197 mimic transfection produced a significant increase in the proportion of apoptotic cells in the 25 nM doxorubicin treatment group, up to the similar level of the 50 nM doxorubicin treatment control group, suggesting a chemosensitizing effect of miR-197 (Fig. 6a, b). In contrast, OCI-Ly1 cells did not exhibit a significant difference in the proportion of apoptotic cells upon transfection with miR-197 mimic in each concentration group of doxorubicin treatment (Fig. 6c, d). Therefore, miR-197 might have an important role in doxorubicin chemosensitivity by enhancing doxorubicin-induced apoptosis in SUDHL9, an ABC DLBCL cells. This finding is consistent with the results from patient samples from the R-CHOP cohort and the ABC subgroup.

## Discussion

Treatment outcome of DLBCL has been considerably improved given the development of combination chemotherapy and the introduction of rituximab. However, approximately 1/3 of patients with DLBCL still experience relapse or refractoriness after standard therapy, eventually exhibiting a very poor outcome [10]. Among the numerous clinical and molecular subtypes of DLBCL, the ABC subtype exhibits a high rate of relapse or refractoriness, and targeting strategies focusing on the ABC subtype are being developed while its clinical utility still remains to be validated [46, 47]. Considering that microRNAs interfere with multiple targets involved in cell survival and drug resistance, we focused on the role of miR-197 in DLBCL and investigated whether it could determine the responsiveness of standard R-CHOP therapy and enhance the sensitivity of doxorubicin in DLBCL cells. In the present study, we observed that (1) miR-197 expression levels were significantly low in the DLBCL progression group, (2) low miR-197 levels were associated with shorter PFS in the R-CHOP cohort and ABC subgroup, and (3) miR-197 enhanced doxorubicin sensitivity in SUDHL9 cells of the ABC subtype.

MiR-197 is involved in apoptosis in solid tumors and hematologic neoplasms. In lung cancers, miR-197 suppresses apoptosis in a p53-dependent manner, playing an oncogenic role [40]. In contrast, a different role of miR-197 in apoptosis is noted in hematologic cells. In myeloma cells, miR-197 and miR-137 induce apoptosis by targeting Mcl-1 [41]. The researchers focused on frequent loss of the 1p12-21 region containing miR-137/miR-197 genes and demonstrated reduction of Mcl-1 protein and induction of apoptosis upon transfection of miR-137/miR-197 into myeloma cells as well as in vivo tumor regression by intratumoral injection of miR-137/miR-197 in a xenograft murine model of myeloma. The inhibiting effect of miR-197 to Mcl-1 may also be at least partly effective in ABC DLBCL because gain or amplification of the Mcl-1 gene was noted in 26% of ABC DLBCL [48]. ABC DLBCLs are thought to be derived from B cells at a plasmablastic stage frequently expressing genes of mature plasma cells [49, 50] and exhibit CBM complex (CARD11, BCL10, and MALT1)-driven constitutive activation of NF-κB signaling [51, 52], which is a key oncogenic signaling pathway that activates Mcl-1 in multiple myeloma [53].

Another important oncogenic signaling pathway in ABC DLBCL is the cytokine signaling/Janus kinase–signal transducer and activator of transcription (JAK–STAT) pathway [49]. A study using ABC DLBCL cells revealed that aberrant expression of IL-22 receptor 1 and IL-22 stimulation increased STAT3 and ERK1/2 activation, suggesting the important pathogenic roles of the IL-22/IL-22R1/STAT3 pathway in ABC DLBCL [54]. Remarkably, miR-197 reduces STAT3 signaling via miR-197/IL-22/STAT3 and miR-197/IL-6/STAT3 pathways in human keratinocytes and hepatocellular carcinomas [55, 56]. In addition, MyD88 mutations occur in > 30% of ABC DLBCL cases and activate both NF-κB and JAK–STAT signaling pathways [57], both of which participate in anti-apoptosis, cell survival and chemoresistance [49, 58, 59].

DLBCL exhibits heterogeneous clinical outcomes and molecular alterations [49]. The wide distribution of miR-197 expression levels in our data may reflect the heterogeneous molecular nature of DLBCL and its ABC subtype. Considering that microRNAs constitute complex multiple network of signaling pathways [60], relative expression levels of miR-197 may indicate inhibition of NF-κB/Mcl-1 and STAT3 signaling in ABC DLBCL, suggesting the possible role of miR-197 as a biomarker of progression in DLBCL, especially in the ABC subtype.

DLBCL heterogeneity was also demonstrated in hierarchical clustering analysis of microRNA chip-based expression profiling, where the cases were not clustered based on disease type or EBV status. This finding may partly be associated with the general cancer-related but not lymphoma-specific properties of the microRNA panel and the relatively small number of cases analyzed. Based on this type of screening, we observed that a miR-197 was distinctively identified and was associated with lymphoma progression and chemoresistance, which could be a cancer-related property, as demonstrated in the clinicopathologic analysis with clinical samples and the in vitro assays.

In vitro studies using the SUDHL9 cell line of the ABC subtype revealed that the effect of low-level (25 nM) doxorubicin treatment was approximately equivalent to treatment with twice (50 nM) the concentration. This effect might suggest the possibility of miR-197 as a chemosensitizing adjuvant agent to reduce doxorubicin concentrations, which might provide the same tumor-killing effect and reduce the cardiac toxicity associated with doxorubicin. This strategy to supplement miR-197 requires further clarification with pre-clinical and clinical studies.

In our in vitro investigation, we focused on the role of miR-197 in doxorubicin sensitivity because doxorubicin is an essential component of chemotherapeutic regimens that kill aggressive B lymphoma cells, including the standard R-CHOP regimen as well as R-EPOCH (rituximab, etoposide, prednisone, vincristine, cyclophosphamide and doxorubicin) and hyper-CVAD (course A: cyclophosphamide, vincristine, doxorubicin, and dexamethasone; course B: methotrexate and cytarabine) [61]. Moreover, sufficient doses of doxorubicin are critical for clinical outcome in DLBCL patients [62]. It still remains to be clarified further whether miR-197 may also influence the effects on sensitivity of other drugs.

## Conclusions

Taken together, the results in our study suggests the clinicopathologic implication of miR-197 in DLBCL. Low miR-197 expression is associated with frequent progression and reduced PFS in DLBCL after standard R-CHOP therapy, especially in the ABC subtype. In vitro studies reveal a chemosensitizing effect of miR-197 in SUDHL9 cells of the ABC subtype treated with doxorubicin. These findings suggest a possible role of miR-197 as a useful biomarker to predict progression and as a chemosensitizer to enhance therapeutic efficacy, providing insight into effective therapeutic strategies with detailed risk assessment in DLBCL.

## Additional file

**Additional file 1: Fiugre S1.** Hierarchical clustering of diffuse large B cell lymphoma cases and microRNA expression profiling with complete agglomerative (a), single agglomerative (b), Ward method (c), and divisive method (d).

## Abbreviations

DLBCL: diffuse large B cell lymphoma
GCB: germinal center B-like
ABC: activated B cell-like
R-CHOP: rituximab plus cyclophosphamide, doxorubicin, vincristine, and prednisone
IPI: international prognostic index
FFPE: formalin-fixed paraffin-embedded
LDH: lactate dehydrogenase
CBM complex: CARD11, BCL10, and MALT1 complex
JAK–STAT: Janus kinase–signal transducer and activator of transcription
