# Supplementary figures and images for: Clinicopathologic implication of microRNA-197 in diffuse large B cell lymphoma
Source: J Transl Med. 2018 Jun 11;16:162. doi: 10.1186/s12967-018-1537-0 (PMC5996506; doi:10.1186/s12967-018-1537-0)

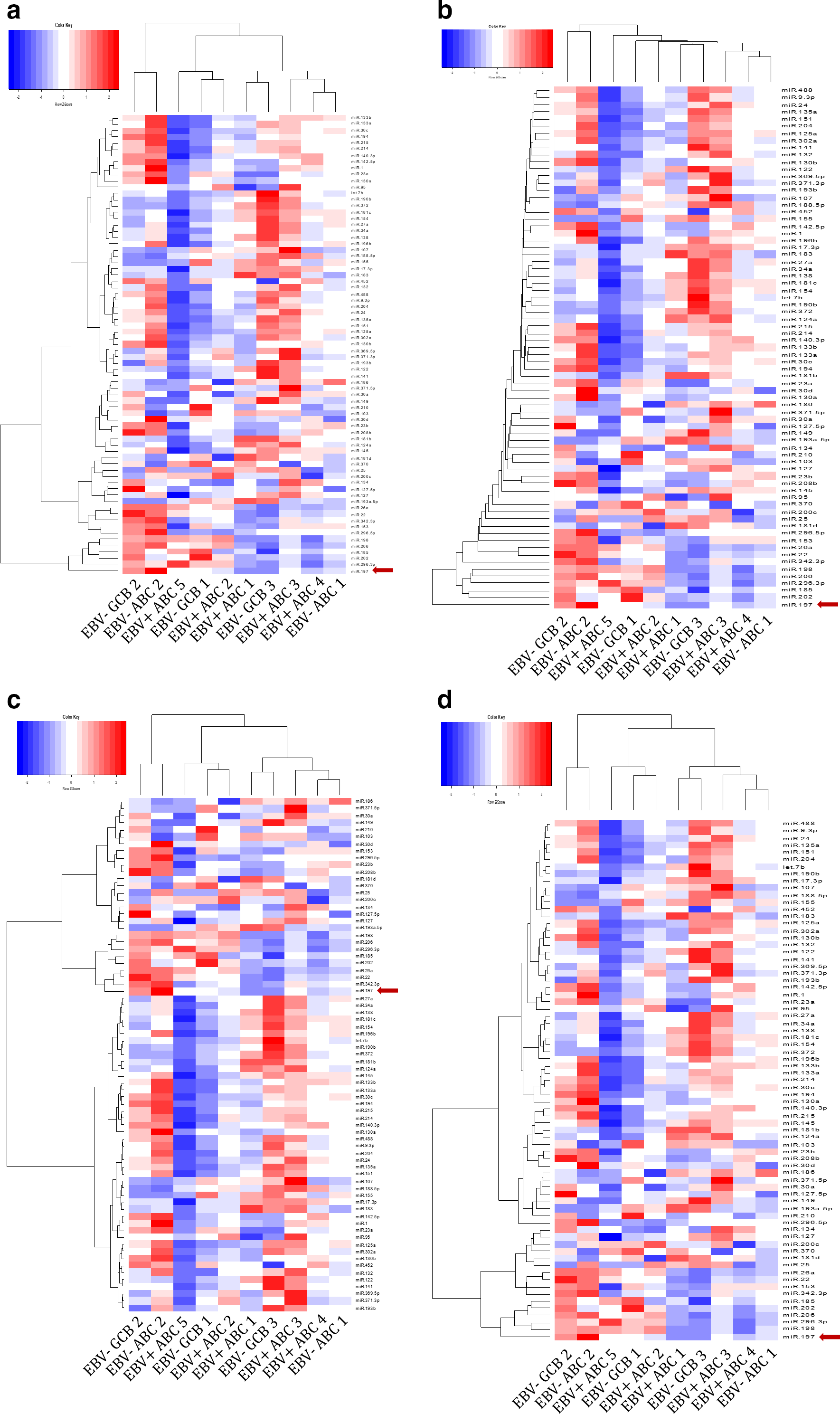

Supplement: Supplementary file 1 — Additional file 1: Fiugre S1. Hierarchical clustering of diffuse large B cell lymphoma cases and microRNA expression profiling with complete agglomerative (a), single agglomerative (b), Ward method (c), and divisive method (d). [file 12967_2018_1537_MOESM1_ESM.tif]
